# All Benign and Malignant Apocrine Breast Lesions Over-Express Claudin 1 and 3 and Are Negative for Claudin 4

**DOI:** 10.1007/s12253-019-00662-9

**Published:** 2019-05-01

**Authors:** Sami Shousha, Oliver Anscombe, Taneisha McFarlane

**Affiliations:** 1 Department of Histopathology, Charing Cross Hospital and Imperial College, Fulham Palace Road, London, W6 8RF UK; 2 Present Address: Royal School of Mines, Imperial College, London, SW7 2AZ UK

**Keywords:** Breast, Apocrine breast carcinoma, Triple negative carcinoma, Claudin 1, Claudin 3, Claudin 4

## Abstract

Invasive apocrine carcinoma of the breast is an uncommon triple negative tumour that lacks a specific therapeutic target. Apocrine metaplasia of the breast shares common morphological features with apocrine carcinoma, and was previously found to consistently over-express claudin 1 and to lack claudin 4. This study was aimed at finding whether apocrine carcinoma, and other related apocrine breast lesions, have similar claudin profile. The immunohistochemical expression of claudin 1, 3 and 4 was studied in 11 cases of in situ and invasive apocrine breast carcinoma, 7 benign apocrine lesions and 45 consecutive morphologically non-apocrine triple negative breast carcinomas. All cases were also immunostained for Gross Cystic Disease Fluid Protein-15 (GCDFP-15), a marker for apocrine differentiation. Apocrine breast lesions maintained their expression pattern from benign through DCIS to invasive carcinoma; all showing strong expression of claudin 1 and 3 and absence of claudin 4. The same pattern of expression was seen in 2 out of the 45 morphologically non-apocrine tumours, but both showed strong positive staining for GCDFP-15. It is concluded that all benign and malignant apocrine lesions of the breast have a consistent pattern of claudin 1, 3 and 4 expression, suggesting the presence of a specific pathway for the development of invasive apocrine carcinoma. The over-expression of claudin 1 and 3 may have therapeutic implications as targets for managing apocrine cancers.

## Introduction

The claudins are a family of small transmembrane proteins that constitute the integral proteins of the tight junctions between epithelial cells that maintain cellular polarity, mediate permeability and are involved in signalling between the cells and their environment [1, 2]. Most tissues or cell types express multiple claudins which are specific for a given tissue or cell [1]. Altered expression of several claudins, in particular claudin 1, 3, 4 and 7 has been linked to the development of various cancers [1], and are thought to be implicated in the metastatic process [3]. The alteration can be in the form of over-expression or down regulation depending on the type of cancer [4].

In benign breast tissue, claudin 1, 3 and 4 were detected by immunohistochemistry in the membranes of the majority of normal duct cells [5]. Cells showing apocrine metaplasia were particularly positive for claudin 1 and consistently negative for claudin 4 [5]. To the best of our knowledge, claudin expression in other apocrine breast lesions has not been reported.

In breast carcinoma, down-regulation and up-regulation of the various claudins occur even in the same class of tumours [3, 5–16]. In triple negative breast carcinoma, claudin 1 has been reported to be expressed in 44.5% of cases [17], compared with 5% in ER positive tumours [7]. Claudin 1 expression correlates with better prognosis and lower rates of recurrence and lymph node metastasis [17, 18]. As Claudin 1 is a trans-membrane protein with 2 large extra-cellular loops, it has been suggested that it could be a candidate for use in therapeutic strategies [10].

Claudin 3 expression has been demonstrated in 79% of ER negative tumours and 89% of ER positive tumours [7]. Claudin 4 is expressed in 79% of ER negative tumours and 52% of ER positive tumours [7] and it is particularly high in basal-like tumours [8]. Increased claudin 4 expression is associated with poor prognosis, high tumour grade and lymph node and distal metastasis [8, 9, 19]. Claudin 4 expression was found to be inversely related to androgen receptor expression [19]. Claudin 3 and 4 are expressed in Paget’s disease of the nipple [20]. Loss of expression of claudin 3 and 4 promotes epithelial-mesenchymal transition [21].

This study was aimed at investigating claudin expression in benign and malignant apocrine breast lesions and compare this with the expression in morphologically non-apocrne triple negative carcinomas as well as in a variety of non-apocrine benign breast lesions.

## Materials & Methods

A total of 129 formalin fixed, paraffin –embedded tissue samples were studied. These included 117 excision, and 12 core biopsies. The diagnosis of benign and malignant apocrine lesions was based on the characteristic eosinophilic staining of the cytoplasm and was confirmed by immunostaining for Gross Cystic Disease Fluid Protein-15 (GCDFP-15) which showed strong diffuse expression of the protein in the majority of tumour cells [22]. All 45 morphologically non-apocrine tumours were also stained for GCDFP-15. All invasive carcinomas, apocrine or non-apocrine, were ER, PgR and HER2 negative, although 2 apocrine cases showed week HER2 positivity.

Tissue blocks were obtained via the Charing Cross Hospital surgical databases (SunquestCoPathPlus™ 2007). The histopathological diagnoses of the breast tissue used are detailed in Tables 1. Ethical approval was granted by Imperial College Healthcare NHS Trust Tissue Bank on 02/08/2015, Project number R15039.

**Table 1:** A list of the cases studied and the result of their claudin staining

Diagnosis	Number of cases	Claudin 1	Claudin 3	Claudin 4
Normal	5	–	+	+
Columnar cell change	5	–	+	–
Usual type ductal hyperplasia	3	–	–	–
Sclerosing adenosis	1	+	–	+
Fibroadenoma (epithelial elements)	5	–	+	+
Tubular adenoma	5	+	+	+
Phyllodes tumour (epithelial elements)	5	+	+	+
Radial scar	2	+	+	+
Intraduct papilloma	5	–	+	+
Flat epithelial atypia	1	–	+	+
Atypical ductal hyperplasia	2	+	+	+
In situ lobular neoplasia	5	–	–	–
DCIS, non-apocrine	14	–	−/+	−/+
Apocrine cyst	1	+	+	–
Apocrine adenosis	6	+	+	+
Apocrine DCIS	5	+	+	–
Invasive apocrine carcinoma	6	+	+	–
Toker Cells	4	–	–	–
Paget’s disease of the nipple	4	–	–	+
Invasive triple negative non-apocrine carcinomas	45	See Table 2		

- Negative, + weekly positive, + strongly positive

Paraffin tissue blocks were cut into 4 μm sections using a Rotary Microtome H325 (Leica Biosystems™). A total of 4 sections were cut per tissue block and floated on water at 40 °C before being loaded onto Superfrost™ Plus IHC microscopy slides (Thermo© Scientific). One slide was stained with H&E and 3 used for immunohistochemistry using the following antibodies: Rabbit anti claudin 1 pAb (ab15098) (Abcam™), Rabbit anti claudin 3 pAb (34–1700) (LifeTechnologies™) and mouse anti claudin 4 mAb (32–9400) (LifeTechnologies™). Antibodies were prepared with Bond Primary Antibody Diluent (AR9352) (Leica Biosystems™). IHC was performed using a Bond Polymer Refine Detection Kit (DS9800) (Leica Biosystems™) of which the constituents are as follows: 4% hydrogen peroxide block (30 ml), post primary rabbit anti mouse IgG (<10 μg/ml) in 10% animal serum in tris-buffered saline (30 ml), polymer anti-rabbit poly-HRP-IgG in 10% animal serum (30 ml), 66 mM 3,3′-Diaminobenzidine tetrahydrochloride hydrate (DAB) chromogen (62.4 ml) and haematoxylin counterstain (30 ml).

Slides were heated at 60 °C for 20 min, promoting tissue adhesion, before being deparaffinised in two changes of xylene for 5 min each and hydrated in 3 changes of 100% ethanol for 5 min each, and cold water for 5–10 min. Heat-mediated antigen retrieval was achieved via steam-heating the tissues in hot citrate buffer (10 mM sodium citrate, 0.05%, pH 6.0) in a steam heater for 30 min. Slides were then allowed to cool down in running water. The tissues underwent a PBS wash followed by hydrogen peroxide for 10 min, before primary antibodies (claudin 1, 3, or 4) were added (200 μl/slide) and allowed to incubate for 1 h. The slides were then washed again with PBS and incubated with post primary antibody for 20 min, followed by polymer for 30 min. Visualisation of the Antibody/antigen reaction was achieved via the Bond DAB chromogenic detection kit and the slides were finally counterstained with hematoxylin (Leica Biosystems™). Slides were cover-slipped automatically using a Leica CV5030 machine.

## Results (Tables 1 & 2)

The staining results were expressed as negative (−), where no positive membrane staining was seen, weekly positive (+) where there was faint usually localised membrane staining (similar to 1+ HER2 expression) and strongly positive (+) where there was strong membrane staining of the majority of cells (similar to 3 + HER2 expression).

**Table 2:** Claudin staining results for invasive non-apocrine triple negative tumours

Diagnosis	Number of cases	–	+	+
Claudin l	45	32 (71%)	7	6
Claudin 3	44	36 (82%)	6	2
Claudin 4	44	17 (39%)	16	11

- Negative, + weekly positive, + strongly positive

**Fig. 1:**
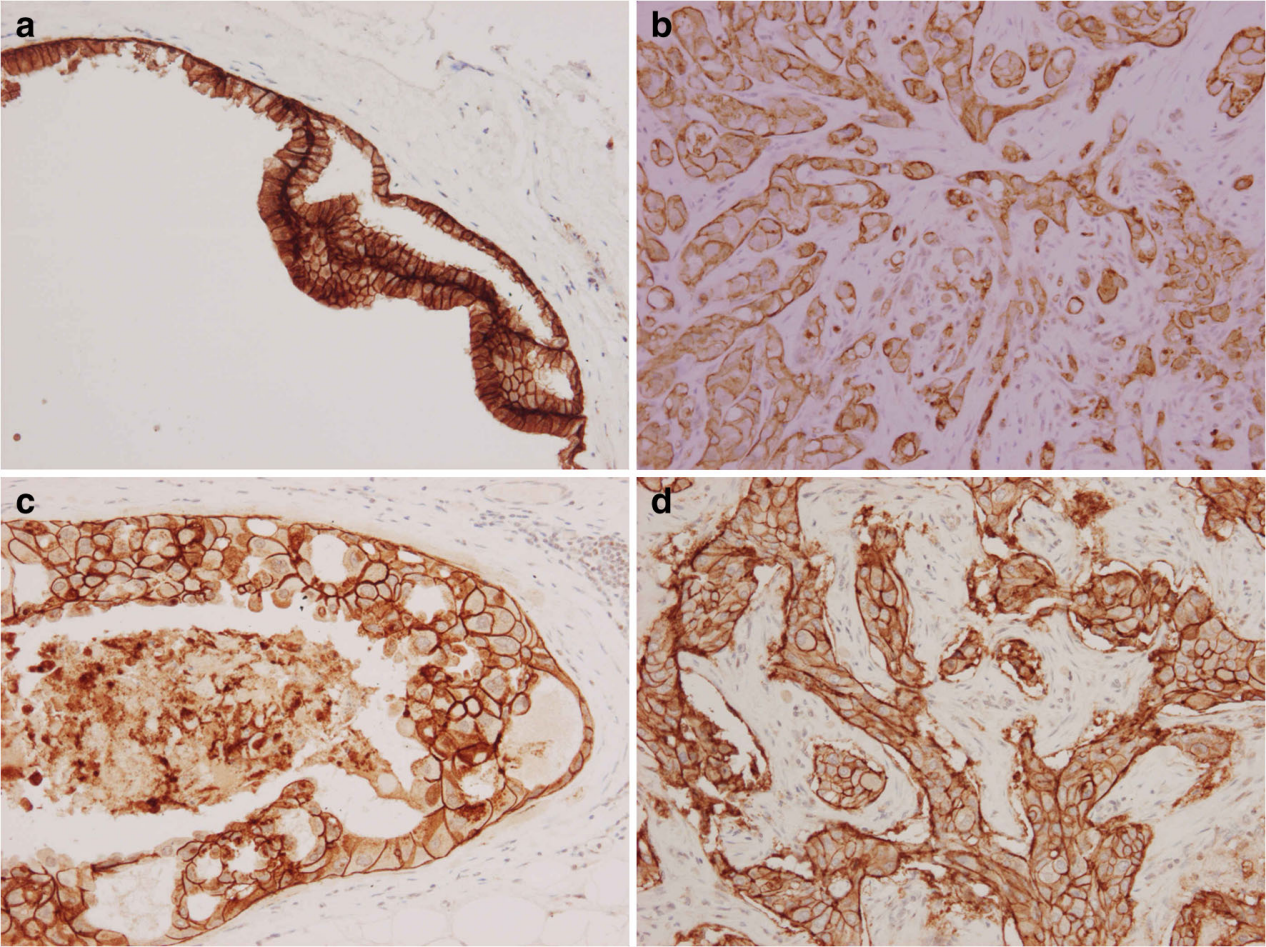
Expression of claudin 1 in apocrine breast lesions: (**a**) Simple apocrine cyst (**b**) Apocrine adenosis (**c**) Apocrine DCIS (**d**) Invasive apocrine carcinoma (immunoperoxidase stain)

**Fig. 2:**
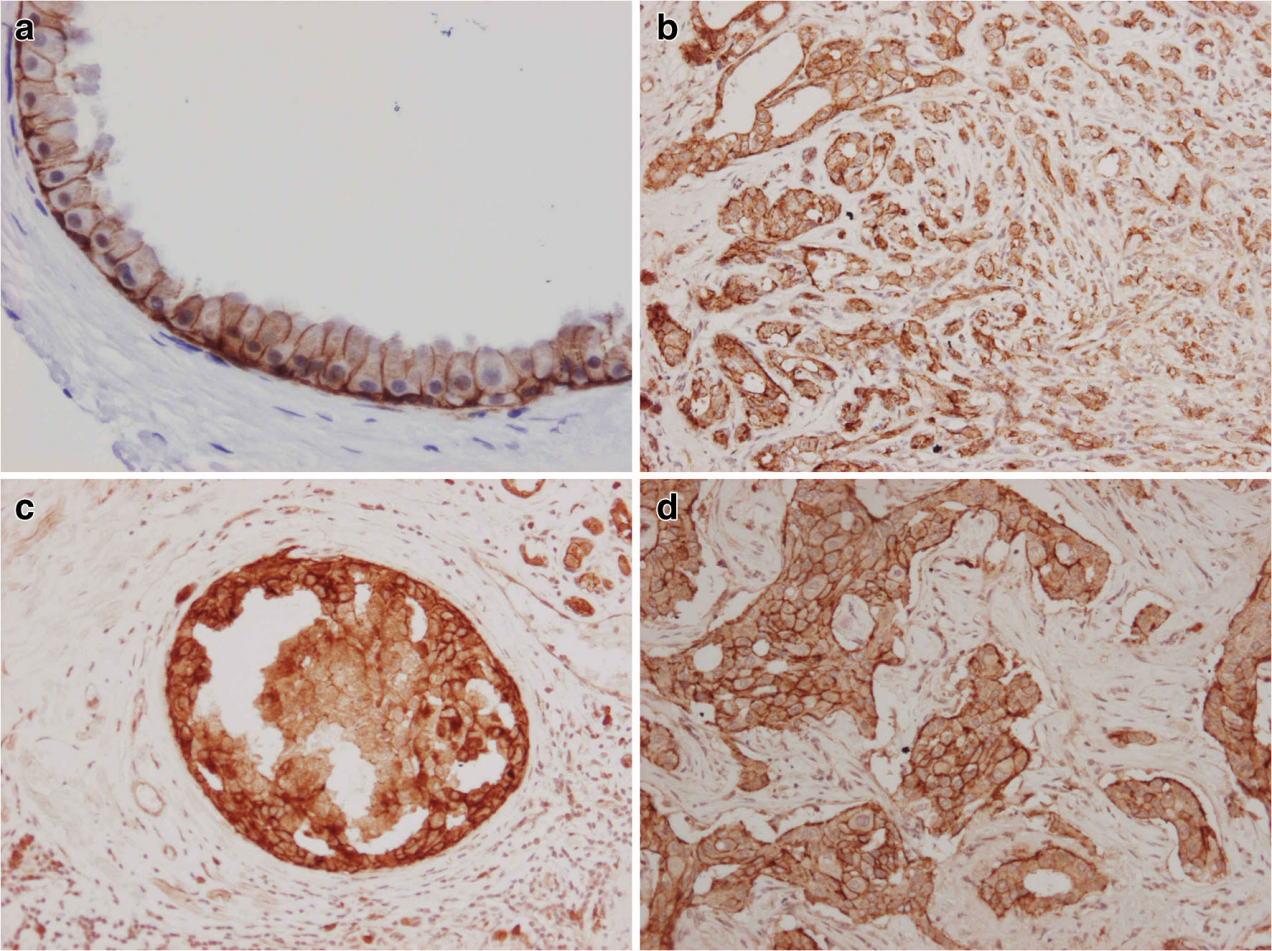
Expression of claudin 3: (**a**) Simple apcrine cyst (**b**) Apocrine adenosis (**c**) Apocrine DCIS (**d**) Invasive apocrine carcinoma (immunoperoxidase stain)

**Fig. 3:**
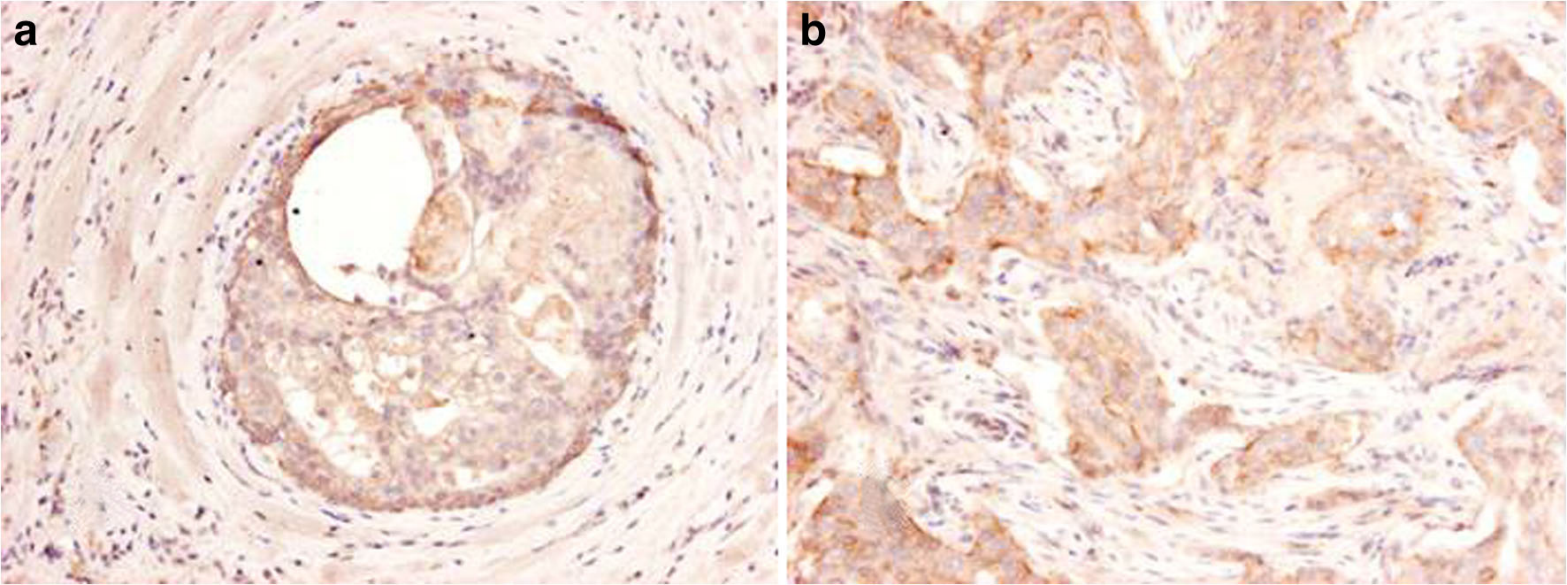
Expression of claudin 4 in neoplastic apocrine breast lesions: (**a**) Apocrine DCIS (**b**) Invasive apocrine carcinoma (immunoperoxidase stain)

Apocrine breast lesions maintained their expression pattern from benign through to DCIS and invasive carcinoma; all showing strong expression of claudin 1 (Fig. 1) and 3 (Fig. 2) and absence of claudin 4 (Fig. 3). This pattern was not seen in any of the non-apocrine benign or DCIS lesions, but was seen in 2 out of the 45 morphologically non-apocrine triple negative tumours. However, both tumours were GCDFP-15 strongly positive. On the other hand, three GCDFP-15 negative cases showed strong Claudin 1 positivity: one was a lympho-epithelioma-like carcinoma, the second was an invasive ductal carcinoma with abundant tumour infiltrating lymphocytes and the third had features of micropapillary carcinoma. All 3 cases were also positive for claudin 4, and two were positive for claudin 3. The majority of the non-apocrine triple negative tumours were negative for claudin1 (71%) and claudin 3 (82%), and weakly or strongly positive for claudin 4 (61%; Table 2). Cases of non-apocrine DCIS were negative for claudin 1 and negative or weakly positive for claudin 3 and 4.

## Discussion

To the best of our knowledge, this is the first study of claudins in a wide range of apocrine breast lesions. We found a consistent expression pattern from benign to invasive carcinoma. All cases examined including benign apocrine cysts, apocrine adenosis, apocrine DCIS and invasive apocrine carcinoma showed strong expression of claudins 1 and 3, and almost complete absence of claudin 4. This combination was not noted in any of the other non-apocrine benign and in situ neoplastic lesions examined and was only found in 2 cases of morphologically non-apocrine triple negative tumours that were GCDFP-15 strongly positive.

Invasive apocrine carcinomas are a distinct subgroup of triple negative breast carcinoma [23]. They have more favourable prognosis and low propensity to metastasise than other triple negative tumours [24]. This fact is consistent with the claudin findings in this study. Claudin 1expression has been shown to be able to exert tight junction- mediated gate function in tumour cells even in the absence of other tight junction-associated proteins [25]. Claudin 1 over-expression in breast carcinoma correlates with better prognosis and lower incidence of axillary lymph node metastasis [17, 18]; while its decreased expression is associated with higher incidence of recurrence and lymph node metastasis and short disease free interval [18]. Claudin-1 expression in non-small cell lung carcinoma is a good prognostic factor [26]. This led to the suggestion that claudin 1 may be used as a therapeutic target for tumours over-expressing it [10].

The finding of absent expression of claudin 4 in apocrine carcinoma, with its known good prognosis, is also consistent with the reported association between claudin 4 over-expression and poor prognosis and increased incidence of axillary and distant metastasis in breast carcinoma [8, 9, 19]. The finding is also consistent with the reported presence of an inverse relationship between claudin 4 and androgen receptor expression [19], as apocrine carcinomas are usually androgen receptor positive [22, 24].

The findings concerning the consistent pattern of claudin expression in all apocrine lesions of the breast, suggest the presence of a specific pathway for the development of invasive apocrine carcinoma. The over expression of claudin 1 and 3 in these triple negative tumours may be worth considering as possible therapeutic targets. In this context, affecting other benign apocrine lesions that might be present in the breast at the same time, ought not to be of concern, particularly as some of these lesions; e.g. atypical apocrine adenosis, may have a pre-cancerous potential [22].
